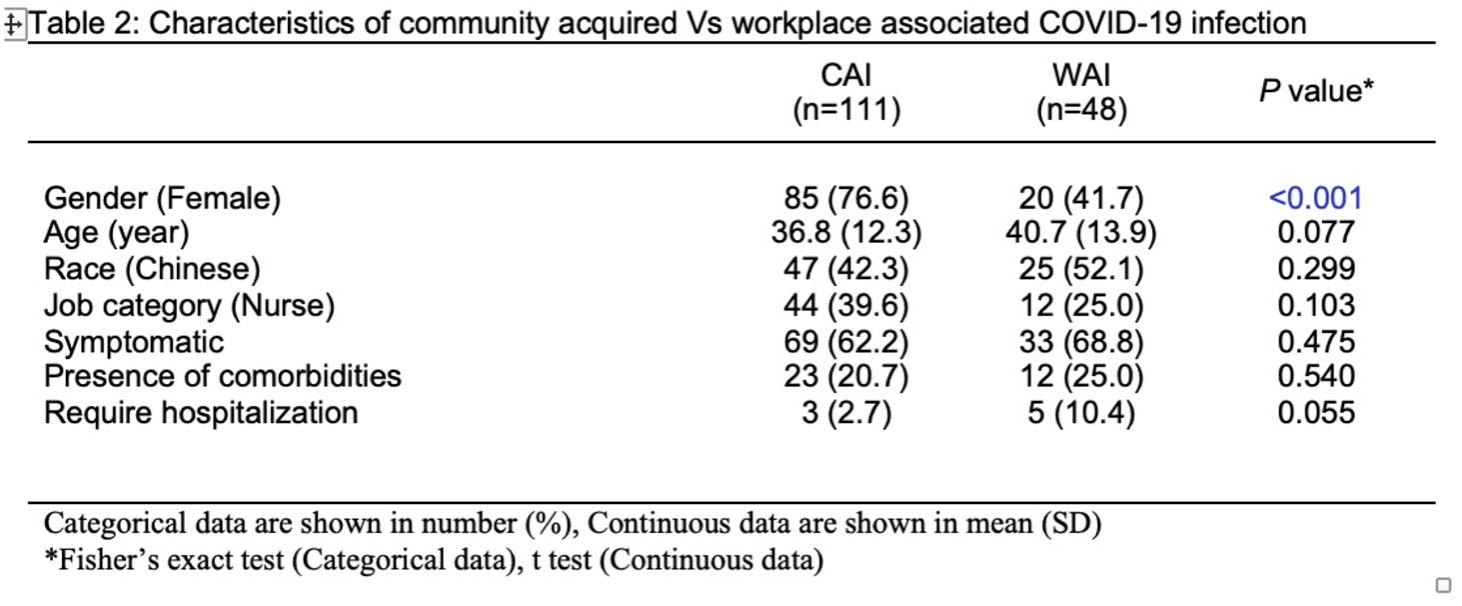# COVID-19 among healthcare workers of a tertiary-care hospital in Singapore

**DOI:** 10.1017/ash.2022.120

**Published:** 2022-05-16

**Authors:** Aung Myat Oo, Indumathi Venkatachalam, Xiang Ying Jean Sim, May Kyawt Aung, Conceicao Edwin Philip, Yang Yong, Shalvi Arora, Shawn See Wee Jin, Wai Leong Lester Lee, Fong Yuke Tien, Lai Chee Lee, Moi Lin Ling

## Abstract

**Background:** Singapore General Hospital (SGH) is the largest acute tertiary-care hospital in Singapore. Healthcare workers (HCWs) are at risk of acquiring COVID-19 in both the community and workplaces. SGH has a robust exposure management process including prompt contact tracing, immediate ring fencing, lock down of affected cubicles or single room isolation for patient contacts, and home isolation orders for staff contacts of COVID-19 cases during the containment phase of the pandemic. Contacts were also placed on enhanced surveillance with PCR testing on days 1 and 4 as well as daily antigen rapid tests (ARTs) for 10 days after exposure. Here, we describe the characteristic of HCWs with COVID-19 during the third wave of the COVID-19 pandemic. **Methods:** This retrospective observational study included all SGH HCWs who acquired COVID-19 during the third wave (ie, the 18-week period from September 1 to December 31, 2021) of the COVID-19 pandemic. Univariate analysis was used to compare characteristics of work-associated infection (WAI) and community-acquired infection (CAI) among HCWs. **Results:** Among a workforce of >10,000 at SGH, 335 HCWs acquired COVID-19 during study period. CAI (exposure to known clusters or household contact) accounted for 111 HCW infections (33.1%). Also, 48 HCWs (14.3%) had a WAI (ie, acquired at their work places where there was no patient contact). Among WAsI, only 5 HCWs had hospital-acquired infection (confirmed by phylogenetic analysis). The sources of exposure for the remaining 176 HCWs were unknown. Weekly incidence of COVID-19 among HCWs was comparable to the epidemiology curve of all cases in Singapore (Fig. 1 and 2). The mean age of HCWs with COVID-19 was 39.6 years, and most were women. At the time of positive SARS-CoV-2 PCR test, 223 HCWs were symptomatic, and 67 (20.0%) of them had comorbidities. Only 16 HCWs (4.8%) required hospitalization, and all recovered fully with no mortality (Table 1). Being female was associated with community COVID-19 acquisition (OR, 4.6, P **Conclusions:** During the thrid wave of the COVID-19 pandemic, a higher percentage of HCWs at SGH acquired the infection from the community than from the workplace. Safe management measures, such as universal masking, social distancing, and robust exposure management processes including prompt contact tracing and environmental disinfection, can reduce the risk of COVID-19 in the hospital work environment.

**Funding:** None

**Disclosures:** None

**Figure:**
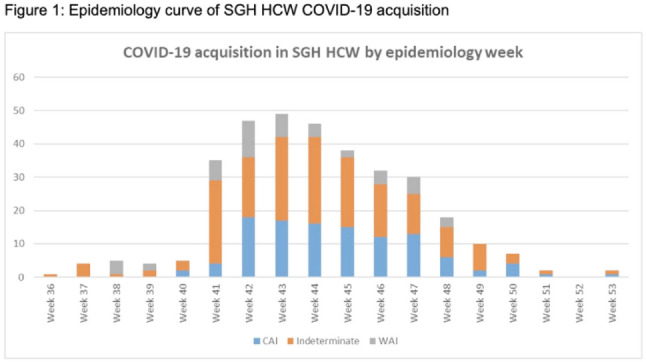

**Figure:**
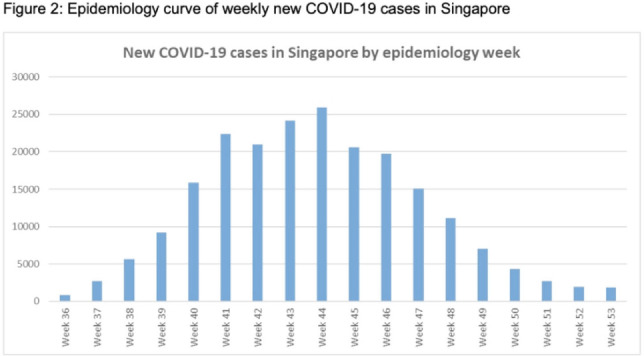

**Table:**
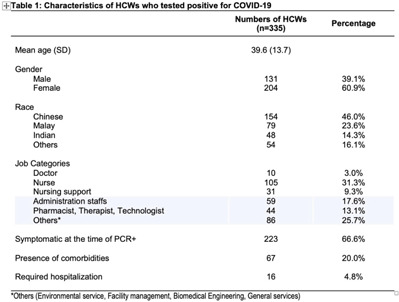

**Table:**